# Teachers’ Commitment and Self-Efficacy as Predictors of Work Engagement and Well-Being

**DOI:** 10.3389/fpsyg.2022.850204

**Published:** 2022-04-26

**Authors:** Kunyao Shu

**Affiliations:** North China University of Water Resources and Electric Power, Zhengzhou, China

**Keywords:** positive psychology, well-being, engagement, self-efficacy, teacher commitment

## Abstract

In the field of language-education psychology, the psychology of educators was not at the center of attention to some extent, despite the immense attention given to the psychology of students. Recently, positive psychology has become very important as it puts more emphasis on the constructive dimensions of life and it regards individual well-being as a major problem for individuals’ achievement. Since the core of an instructing institution is the educators, their well-being must be prioritized in the academic circumstances to both improve students’ skills and to motivate and inspire participation and involvement in the class. Moreover, teacher engagement is viewed as another significant factor in this realm that refers to people’s outlook toward their job, impacting their emotional attendance and engagement during their presentation. Also, there is an inner force that pushes educators to put more time and strength in maintaining participation in the school that is called educator commitment that is determined on evolving the school builds an emotive connection between educators and schools. It eventually encourages educators to improve their instructing careers and find ways to create a successful educational setting that would enable learners to attain their goals. In addition, teacher self-efficacy, including educators’ convictions in their skill to successfully manage assignments, responsibilities, and difficulties associated with their expert activity, has an important function in affecting key scholastic results in the career setting. By taking the aforementioned constructs, this review of literature provides implications for academics, teachers, and policymakers in search of better considering the functions of commitment and self-efficacy on their work engagement and well-being.

## Introduction

As a career, teaching is regarded demanding, challenging, and stressful (Liu and Onwuegbuzie, 2012), and the conviction that a well-working educator is a better educator in dealing with stress gives rise to the concern of teacher well-being and it is an issue that has comprehensively been inspected within the scope of stress and exhaustion or in terms of commitment (Spilt et al., 2011; Zee and Koomen, 2016). Teacher well-being has gained the attention of both researchers and practitioners over the last years by the arrival of Positive Psychology (PP) (Seligman and Csikszentmihalyi, 2014). Positive Psychology emphasizes perfect functioning concerning physical, societal, psychological, and emotive well-being and to the constructive qualities of individuals, it refers to the positive perceptions and emotions, the constructive function of the setting and organizations in the development of a person’s well-being (Dewaele et al., 2019; Wang et al., 2021). New research has examined and outlined the positive and negative elements associated with well-being predictors, like exhaustion, work fulfillment, and constructive/deconstructive effects (Pretsch et al., 2012). Teacher well-being is regarded as crucial for personal educators and the entire academic framework and it is broadly maintained to have an important function in the standard of educators’ lives, learners’ well-being, and the solidity of the instructing employees (Acton and Glasgow, 2015). Indeed, teacher well-being, associated with exemplary psychological competence and their constructive work experience, is described by the presence of constructive aspects like career satisfaction and job interest (Benevene et al., 2018). Even though the well-known needs of instruction have resulted in the exploration of deconstructive dimensions of educators’ feelings like anxiety and exhaustion, scholars are also curious about how constructive encouraging elements, like engagement, are built and promoted in different school environments (Benevene et al., 2018). Indeed, it is related to the connection between the worker and his/her job and it is theoretically different in itself from the commitment to the function or employer, from the fulfillment attained from that job, and from the willingness to stay or resign (Schaufeli, 2013). Work engagement is characterized as a constructive, satisfying, career-relevant mental condition that is described by vigor, dedication, and absorption (Schaufeli, 2013). In the last 10 years, it has attained significant study attention in common workplace environments and it is related to efficiency and involvement in the workplace, this means that engaged educators are more prone to add to school life and assume more tasks outside the class (Parker and Martin, 2009). Engagement in a scholastic setting usually alludes to learners’ diligent participation in education, the standard of endeavor, and the degree to which the setting is successfully utilized to facilitate education (Krause and Coates, 2008). Because educators’ engagement is a determinant of learners’ success and is also related to educators’ productiveness, scholars and academic legislators are beginning to focus more on it. Therefore, it can be inferred that engaged educators are unlikely to leave their careers (Bakker and Bal, 2010). Well-being and engagement are conventionally comprehended as job fulfillment and pleasure, and joy in the corporate culture (Richardson and Watt, 2006).

Moreover, involvement in the workplace is an especially significant factor of engagement for employers. This refers to the extent to which a worker is associated with the life of the institution or accepts additional responsibilities aside from their main ones (Saks, 2006). Undeniably, workplace involvement has been demonstrated to be constructively linked to a commitment to education and deconstructively linked to emotive withdrawal and weak recognition of the workplace. Teachers have their special personality traits, systems of belief, and cognition, which significantly affect their decisions and activities within the class (Derakhshan et al., 2020; Nayernia et al., 2020). As a consequence, assuming that educators’ presentation in their profession is affected by multiple psychological attributes, namely, self-efficacy can be reasonable. The conception of self-efficacy develops from the principle of social cognition which focuses on the concept that individuals could affect their agency (Bandura, 2006). Self-efficacy is similar to individuals’ ability to act, i.e., it indicates whether a person’s behavior can affect the intended performance and it is not only an effective component with reference to an individual’s goals and behaviors, but also depends on the context in which it occurs (Van Acker et al., 2013). Teacher efficacy relates to their belief in the aptitude of a collection of teachers to take the necessary responsibilities (Han and Wang, 2021). Teacher self-efficacy has a key role in an educators’ choice of personal purposes, the degree of persistence when encountering adversity, and the power of motivation to perform certain behaviors in teaching, like when using learning content of digital teaching (Van Acker et al., 2013; Glackin and Hohenstein, 2018).

Educators with a great level of self-efficacy are more committed and less prone to burnout (Chesnut and Burley, 2015; Fathi et al., 2021). They are inclined to use inventive educational strategies and encounter greater degrees of work fulfillment (Thurlings et al., 2015). Educators are faced with frequent alterations in an academic context like syllabus, teaching, learners’ demands, and academic guidelines (Ganjali et al., 2019). Therefore, to improve learners’ success, educators need to make more endeavors to provide superior instruction. It is worth mentioning that educators’ efforts and participation to enhance superior instruction are mirrored in their commitment to tasks, school, learners, and career, as is individually referenced in the literature (Thien et al., 2014). Furthermore, commitment is of the utmost interest among scholars, out of all the general demeanors associated with work. This is in light of the fact that those who work with great levels of commitment are believed to be more prone to create institutional and personal-level results like employee turnover, presentation, and the desire to stay in or leave an institution (Razak et al., 2010). A circumstance akin to this has been seen in academic environments, where educator commitment is regarded as one of the key elements in the effectiveness of schools and the achievement of academic frameworks (Ganjali et al., 2019) and due to its impactful function in academia, it has attained a great deal of attention around the world. With this in mind, academic researchers have categorized commitment indicators into three classes: individual, institutional, and situational (Price, 2012). Teacher commitment to learners embraces educators’ inclination to assist learners and take accountability for their education. Commitment to instruction offers educators the obligation to examine novel methods of instruction to build learners’ educational encounters. Committed educators are competent to offer learners new teaching techniques that result in greater success (Altun, 2017). Moreover, educators with commitment can develop passionate students by motivating them to participate in the class exercises. Crucial to instructional excellence, educator commitment involves being committed to the school, learners, the continuation of the profession, expert knowledge foundation, and instructional career (Choi and Tang, 2009).

Examining the factors that underline the well-being and engagement of educators may offer some beneficial and valuable results in classifying the antecedents of excellence of education at teaching spaces (Hall-Kenyon et al., 2014; Zee and Koomen, 2016). A bulk of experimental studies has emerged confirming the prominence of self-efficacy in an educational setting (Hampton and Mason, 2003; Klassen and Usher, 2010). Furthermore, educators with more self-efficacy levels are about to involve in specialized programs and employ new educational practices (Kent and Giles, 2017). However, regardless of several inquiries on teacher efficacy and work engagement, to the best of the researcher’s knowledge, not enough attention has been paid to the role of self-efficacy and commitment on their work engagement and well-being.

## Review of the Related Literature

### Self-Efficacy

An individual’s views and opinions on what he or she can do and how well they can do it are referred to as self-efficacy (Zimmerman and Cleary, 2006). From this viewpoint, self-efficacy is regarded as a multi-dimensional concept and as the most core human agency mechanism, i.e., the capability to purposefully affect one’s own functioning and living conditions (Bandura, 2006). Self-efficacy is the belief of people in their aptitude to gain pleasing educational results in an educational setting (Klassen and Usher, 2010), and based on the reports, it is the unique most significant factor in predicting academic results after ability (Wiederkehr et al., 2015). Self-efficacy is a dominant concern in describing how a person acts, reflects, and responds in face of difficult and stressful conditions (Downes et al., 2021). According to the theory of social cognitive, as a multidimensional concept, teacher efficacy is described as their personal view on their capability to plan, establish, and conduct the activities required to accomplish educational purposes (Skaalvik and Skaalvik, 2014). Educator self-efficacy can be broadly characterized as educators’ confidence in the ability to improve learners’ results, have them engaged in the class, have them effectively perform educational assignments, and attain instructional objectives (Hoang and Wyatt, 2021). From an educational point of view, the self-esteem of teachers is viewed as a belief that not only influences teaching and teacher behavior but also influences student learning and behavior (Tschannen-Moran and Hoy, 2001). The efficacy of teachers is related to their beliefs in their ability to perform teaching practices in an educational setting that leads to positive learner results (Lemon and Garvis, 2016). People with self-efficacy are claimed to deliberately select difficult activities, are enthusiastic to spend more time and energy to achieve their goals, and keep on their efforts despite the failure possible in achieving their individual or institutional purposes (Burić and Macuka, 2018).

### Commitment

Commitment has been conceived as a balanced or power that guides manners by limiting freedom and forcing people to adhere to a course of action when confronted with contradicting rationales and demeanors (Meyer and Herscovitch, 2001). Commitment alludes to a demeanor or mental state that describes the connection between a worker with their boss and eventually affects their willingness to remain or leave the institution (Meyer and Herscovitch, 2001; Kotzé and Nel, 2020). Specifically, educator commitment mirrors educators’ feeling of loyalty and attachment to the organization they work at and has been proven to be a significant indicator for various learning and mental results (Day, 2008). According to existing studies, committed educators focus more on their work, emphasize achieving school objectives, and remain in school. Moreover, teacher commitment was discovered to be associated with instructional presentation, absenteeism, burnout, and turnover. It has also been demonstrated to affect learners’ attainment benefits and their performance toward school (Park, 2005). The growth of commitment in the scholastic setting can be logically anticipated to involve active and two-way associations between different psychological, relational, and surrounding elements (Human-Vogel, 2013). Commitment is a cycle with frequent interactions between the individual, professional, and academic structured elements (Choi and Tang, 2009). Due to this frequent interaction, numerous commitments are more essential than others in various circumstances, and the power of such commitments also depends on the consequences of different forces in an individual’s life (Choi and Tang, 2009). Committed educators are involved in communicating with their students and consider their development and they meaningfully struggle for aptitude in cultivating and developing numerous approaches (Day, 2008).

### Work Engagement

Work engagement alludes to a constructive and satisfying career-relevant mental condition described by vitality, devotion, and immersion (Bakker et al., 2011). The feeling of vitality and the conception of the career as an important and purposeful quest is especially highlighted (Bakker et al., 2011). Prior inquiries have confirmed that educator engagement is constructively anticipated by educator self-efficacy and negatively anticipated by emotive fatigue (Salanova et al., 2005). In the same vein, Bakker et al. (2011) predominantly underline the knowledge of vigor and the discernment of the work as a noteworthy and significant issue. Being diligently connected with one’s job and encountering meaning, excitement, and difficulties are known as dedication (Schaufeli and Bakker, 2004). The physical strength of the body or mind at work is known as vigor. Dedication implies an employee’s emotive state of being enthusiastic about their job. Absorption is defined as an intellectual circumstance in which the person is engaged with their work and is entirely focused on it (Bakker et al., 2011). Absorption is explained as being completely rigorous and luckily involved in an individual’s work, whereby time spends hurriedly (Schaufeli and Bakker, 2004). Indeed, the aspect of absorption, associated with being entirely focused on one’s job, is regularly characterized as the quick passage of time or the struggle with leaving work (Mauno et al., 2007). Work engagement is an indicator of inherent encouragement and is associated with constructive results for educators and learners, from a self-determination theory (SDT) point of view (Bakker and Bal, 2010). Engaged educators are stimulated enough to display the vitality and efficiency to finish assignments, and they also can cope with the intricate challenges of everyday work. In line with the assumption of SDT, engagement mirrors an independently controlled type of encouragement that has been proven to bring an improved presentation, perseverance, and inventiveness (Ryan and Deci, 2000). Educators with low engagement during the process of teaching are more prone to sense outer control and are encouraged by the want to receive prizes and keep away from penalizing (Meyer and Gagne, 2008). There are different types of engagement such as cognitive engagement that alludes to the task that is carried out with heed, immersion, and concentration, in which the passage of time is unfelt. Fondness, delight, pleasure, thrill, and entertainment associated with the career of instruction are known as emotive engagement (Han and Wang, 2021). Societal engagement with learners refers to compassion, kindness, and sympathy with learners. Societal engagement with peers hints at a feeling of relation educator experiences with colleagues, placing importance on peer connection, and liking and assisting colleagues (Klassen et al., 2013). Prior research displays that teacher work engagement is significantly anticipated to teacher self-efficacy and negatively anticipated to burnout (Skaalvik and Skaalvik, 2014). Studies also demonstrate that career engagement is related to constructive results, such as less willingness to quit the career of instruction (Bakker and Bal, 2010).

### Well-Being

Especially in the case of the career of instruction, the literature characterizes teacher well-being as a constructive emotive condition as an outcome of the balance between individual demands on the one hand and anticipation of students regarding the school on the other hand (Engels et al., 2004). Well-being is a concept that explains a person’s personal constructive career experience, and it consists of five eudemonic aspects, namely, relational aptitude at work, flourishing at work, sense of ability, discerned acknowledgment at work, and a need for taking part in work (Dagenais-Demarais and Sovoie, 2011). Educator well-being is a constructive emotive condition brought about by a balance between the accumulation of certain contextual elements, individual demands, and anticipations with regard to the school (Engels et al., 2004). Educator well-being is a concept that incorporates an educators’ framework of the standard of their individual, professional, and interpersonal selves (Spilt et al., 2011). The emotional aspect is deemed as a vital element in teacher well-being in an educational cycle (Van Petegem et al., 2005). As stated by Mercer et al. (2020), it has been argued that well-being is indispensable for the actual role of teachers teaching at different levels, since it can boost and foster creativeness and also a constructive relationship with students that increase the learners’ accomplishment. Language teacher well-being received more consideration in educator training programs since their emotional, societal, and qualified well-being meaningfully affect their presentation, classroom setting, and learners’ well-being along with their educational presentation (Mercer, 2020; Greenier et al., 2021).

## Conclusion

Based on the positive role of self-efficacy and commitment, higher education organizations must cultivate and present programs that would develop teacher well-being and engagement in the classroom. It can be concluded that teachers with a moderately low level of commitment usually search for probabilities to resign from their job and have less engagement in the classroom (Shirbagi, 2007). In line with the current review, it can be stated that engagement is viewed as a value of commitment, in other words, as people become committed to their education, their manners must mirror that commitment and help sustain it. People who commit to a specific course of action are alleged to control their manner to sustain that course of action. Teachers with a considerable degree of commitment will be more realistic to capabilities where they work; they will also assist learners’ accomplishment and success effectively.

In addition, the utmost significant factor of career engagement is teacher efficacy since it is associated with the individual skills and abilities that educators can use in their instructing career and all schools or academic settings. In this context, Teacher efficacy gives educators the skill to perfect and enhance instructing strategies, educator-learner, and educator-guardian associations, peer cooperation, decision-making, and the school setting. Teachers who are more self-efficacious are reported to be more involved with learners and have higher satisfaction in their careers (Hampton and Mason, 2003; Granziera and Perera, 2019). They tend more persistent for adversity in teaching and attempt further creative strategies to aid learners comprehend difficult subjects (Zee and Koomen, 2016). Liu and Huang (2019) preserved that teacher efficacy results in educators’ work engagement, contributing to a more constructive educational setting. Since career engagement is related to educator efficiency and learners’ constructive educational results, constructive career engagement is crucial (Lemon and Garvis, 2016; Burić and Macuka, 2018).

Moreover, teachers with a high level of self-efficacy are prone to be better at arranging, more flexible during setbacks, and more unbiased and helpful to their learners. In addition, Fathi and Derakhshan (2019) have maintained that the features of educators and their emotional aspects, namely well-being have a substantial and meaningful impact on the teacher because there is a decisive link between educator well-being, learners’ presentation, and standard of instruction, educators’ well-being is essential to students.

## Implications and Future Directions

Within the scope of the current review, the most noticeable and prominent factors causative to teachers’ engagement as well as well-being are reported. Indeed, the present review concentrated on the role of teachers’ work engagement by the growth in their self-efficacy and commitment. The present review has several implications for EFL educators, educator trainers in which it cares about teachers and scholars in the EFL teaching domain to extend their perspectives on the prominence of teacher commitment and self-efficacy and their functions on their engagement and well-being.

### Implications for Teachers

While there is proof that more degrees of commitment is linked to greater degrees of efficiency and intention of taking on more obligation that lead to more engagement, lower institutional commitment alludes to lower degrees of efficiency, profession flows out, absenteeism, and weak presentation. The more committed educators are in school, the more they become engaged with their job. If academic institutions can make educators more committed, these educators will have a higher chance of being engaged with their job. Therefore, schools need to encourage educators’ commitment to secure more engaged educators. Teachers can benefit from this review in a way to be aware that their high degrees of well-being is about to be effective, more engaged, and they can cope with challenges that take place during their teaching procedure.

### Implications for Faculty Members

Positive Psychology emphasizes a person’s assessable and controllable strong points and mental abilities, as opposed to the weak points like exhaustion, dispute, and discontent with work at the workplace. Thus, institutions today seek active, devoted, and attentive workers; in other words, people engaged in their careers. This is for the reason that such workers are more imaginative and inventive by dedicating their skills and knowledge to the institution (Bakker and Demerouti, 2008). The present review is significant for faculty members and institution managers to be aware that self-efficacious educators are prone to have fewer symptoms of emotive fatigue and exhaustion and have a greater degree of obligation toward instruction, commitment, individual success, and work fulfillment (Zee and Koomen, 2016). Indeed, based on the review of literature, it can be argued that educators with a greater self-efficacy engage more in their job, encounter more enjoyment, honor, and fondness, and lower levels of rage, tiredness, and despair toward their learners (Burić and Macuka, 2018). Furthermore, in the academic framework, teacher well-being is associated with gratitude, purposeful expert growth, and participation in making decisions. For it to be attained, school administrators must have abilities like making connections, contextual skills, as well as societal and emotive skills (Cann et al., 2021). The leadership method could have a constructive impact on work fulfillment by improving employee presentation and corporate objectives through motivation as well as a slow elevation of commitment (Altaf et al., 2019). Regarding the function of teacher efficacy on engagement, the more efficacious teachers are, the more engaged they are in their careers. This fundamentally implies that they are more resolved, devoted, tenacious, lively, encouraged, and excited about their work (Yang, 2021). As a result, faculty managers must provide a vigorous and dynamic situation for their educators to keep and maintain their well-being that can boost a positive and successful classroom. The function of managers must be emphasized to constructively improve educators’ well-being, this is because study evidence demonstrates that constructive and equitable leadership functions of managers affect the well-being of workers (Fathi et al., 2020).

### Implications for Teacher Trainers

Teacher trainers are suggested to include challenging instructing exercises in their educator coaching plans, trigger the execution and coaching of specific and contextual instructional techniques, motivate the suitable utilization of techniques that result in well-being. This is because higher levels of efficacy conceptions in educators have an essential function in enhancing their educators’ well-being (Helms-Lorenz and Maulana, 2016).

Future studies should be conducted to scrutinize associations between the concepts inspected in this paper (teacher commitment, self-efficacy, engagement, and well-being) and demographic factors should be taken into consideration in further research since they will help to extend scholars’ knowledge of how teachers’ gender and experiences relate to the above-mentioned constructs. Briefly, it is similarly prerequisite to associate teacher efficacy and commitment to learners’ accomplishment and examine this association in further studies. Likewise, some empirical research with diverse designs can be carried out to clear out the issue and add to the body of literature.

## Ethics Statement

The studies involving human participants were reviewed and approved by North China University of Water Resources and Electric Power Ethics Committee. The patients/participants provided their written informed consent to participate in this study.

## Author Contributions

The author confirms being the sole contributor of this work and has approved it for publication.

## Conflict of Interest

The author declares that the research was conducted in the absence of any commercial or financial relationships that could be construed as a potential conflict of interest.

## Publisher’s Note

All claims expressed in this article are solely those of the authors and do not necessarily represent those of their affiliated organizations, or those of the publisher, the editors and the reviewers. Any product that may be evaluated in this article, or claim that may be made by its manufacturer, is not guaranteed or endorsed by the publisher.
